# Efficacy of Compound Therapy by Ginseng and Ciprofloxacin on Bacterial Prostatitis 

**DOI:** 10.22074/cellj.2016.3993

**Published:** 2016-04-04

**Authors:** Maryam Miri, Saeid Shokri, Shahram Darabi, Mahmood Alipour Heidari, Akhgar Ghalyanchi, Mohammad Hassan Karimfar, Reza Shirazi

**Affiliations:** 1 Department of Anatomical Sciences, Qazvin University of Medical Sciences, Qazvin, Iran; 2 Department of Anatomical Sciences, Zanjan University of Medical Sciences, Zanjan, Iran; 3 Department of Biostatistics, Qazvin University of Medical Sciences, Qazvin, Iran; 4 Department of Physiology, Faculty of Medicine, Zanjan University of Medical Sciences, Zanjan, Iran; 5 Department of Anatomical Sciences, Zahedan University of Medical Sciences, Zahedan, Iran; 6 Department of Anatomical Sciences, Iran University of Medical Sciences, Tehran, Iran; 7 Cellular and Molecular Research Center, Iran University of Medical Sciences, Tehran, Iran

**Keywords:** Ginseng, Prostate, Infection, Ciprofloxacin

## Abstract

**Objective:**

Genitourinary tract infections play a significant role in male infertility. Infections of reproductive sex glands, such as the prostate, impair function and indirectly affect male fertility. The general aim of this study is to investigate the protective effect of Korean red ginseng (KRG) on prostatitis in male rats treated with ciprofloxacin (CIPX).

**Materials and Methods:**

In this experimental study, we randomly divided 72 two male Wistar rats into 9 groups. The groups were treated as follows for 10 days: i. Control (no medication), ii. Sham [(normal saline injection into the vas deferens and oral administration of phosphate-buffered saline (PBS)], iii. Ginseng, iv. CPIX, v. CIPX+ginseng, vi. Uropathogenic *Escherichia coli* (*E. coli*) (UPEC), vii. UPEC+ginseng, viii. UPEC+CIPX, and ix. UPEC+ginseng+CIPX. The rats were killed 14 days after the last injection and the prostate glands were removed. After sample preparation, routine histology was performed using hematoxylin and eosin staining. The terminal deoxynucleotidyl transferase mediated dUTP-biotin nick end labeling (TUNEL) method was used to determine the presence of apoptotic cells.

**Results:**

The severity score for acinar changes and inflammatory cell infiltration in the UPEC+CIPX group did not significantly different from the UPEC group. However this score significantly decreased in the UPEC+CIPX+ginseng group compared to the UPEC group. Apoptotic index of all ginseng treated groups significantly decreased compared to the UPEC and CPIX groups.

**Conclusion:**

These results suggested that ginseng might be an effective adjunct in CIPX treatment of prostatitis. The combined use ginseng and CIPX was more effective than ginseng or CIPX alone.

## Introduction

Infertility is an important concern among 20% of couples. Approximately 50% of infertility is attributed to males (1,2). Male infertility consists of spermatogenesis disorders, defects of sperm transportation, impotence, hypogonadism and urinary tract infections (UTI) (3,4). Urogenital infections are responsible for approximately 35% of male infertility. These infections may impair accessory gland functions, such as the prostate, and lead to changes in seminal plasma composition (5,6). Therefore, male accessory sex glands infection is a major risk factor for infertility (6). Uropathogenic *Escherichia coli* (*E. coli*) (UPEC) is an important causative agent in more than 70% of urogenital tract infections (7). Antibiotics have long been considered the most effective treatment for bacterial infections. Ciprofloxacin (CIPX), belonging to the family of fluoroquinolones, has a broad spectrum of efficacy for bacterial infections (8). This drug can be transported to the seminal fluid and directly affect sperm cells by reducing sperm concentration, motility and viability (9). In a study, CIPX administration (5 mg/kg body weight) to 70 adult male Wistar rats has resulted in acinar changes, lymphocytic infiltration and fibrosis in the interstitial space in the prostate gland (10). A decrease was observed in testis, epididymis and seminal vesicle weights after administration of CIPX to rats for over 60 days (9). This antibiotic induces oxidative damage in rats and increases reproductive toxicity (11). It can activate caspase 3 and increase the apoptosis process in male germ cells (8). 

Both infections and fluoroquinolones can induce the generation of a tremendous amount of reactive oxygen species (ROS) (12,14). Excessive generation of free radicals can damage proteins, lipids, and nucleic acid structures, in addition to contributing to cellular dysfunction and death (15). According to previous reports, antioxidants can protect against CIPX by eliminating ROS generation during administration of this antibiotic (14). In another study, the removal of accessory reproductive glands from hamsters has led to increased DNA damage in spermatozoa. This fact suggests that these glands are the main source of antioxidants in seminal fluid (16). 

Collectively, by affecting *E. coli* and CIPX on histological structures of male accessory glands, their function as main source of antioxidant, will be changed. Under these circumstances, administration of antioxidants can be useful. 

Korean red ginseng (KRG), a derivative of Panax ginseng, is considered a very powerful antioxidant to be used to eliminate free radicals. Kim et al. (17) have shown that KRG improved rat testis dysfunction by suppression of superoxide production. In addition to anti-stress and antioxidant activities of KRG, there are also potent pharmacologic actions against cancer and diabetes. Choi et al. (18) have proven that the combination therapy of ginsenoside with CIPX is an effective treatment for chronic bacterial prostatitis (CBP). The use of Panax ginseng extract can promote spermatogenesis and increase serum testosterone, follicle stimulating hormone (FSH) and luteinizing hormone (LH) levels. This normalization of hormone levels may be due to the effect of Panax ginseng on the hypothalamic-pituitary axis (3,19). 

However, less is known about the protective effect of KRG on the apoptosis process and structural changes in prostate gland infections. This study seeks to determine the effective role of KRG on UPEC infection of the prostate in a rat model under treatment with CIPX. 

## Materials and Methods

### Animals

In this experimental study, a total of 72 adult male Wistar rats were provided from Shahid Beheshti University, Tehran, Iran. This study was approved by the Medical Ethics Committee of Qazvin University of Medical Sciences. Rats ranged in age from 2 to 3 months and weighed 200 to 250 g. Before the experiment, animals were maintained for one week under controlled environmental conditions (23˚C and a 12 hour/12 hour dark-light cycle). Food and water were available ad libitum. The hygienic conditions were kept constant throughout the experimental period. 

### Experimental groups and treatment

The rats were randomly divided into 9 groups (n 8): i. Control (no medication); ii. Sham (0.1 ml saline injected into the vas deferens+pH=7.2 phosphate-buffered saline (PBS, Sigma, Denmark) administered by oral gavage once daily during 10 days) (20, 21); iii. KRG (intraperitoneal injection [IP] injection of 15 mg ginseng/kg body weight once daily for 10 days) (22); iv. CIPX (150 mg/kg body weight, Cycin, Shahid Beheshti University, Tehran, Iran) administered orally, once daily during 10 days. CIPX is prepared by solving the tablets in distilled water (23); v. CIPX+ginseng; vi. UPEC M39 standard strain was prepared according to recent studies and 0.1 ml (1×10^8^ CFU/mL) was injected into each vas deferens (23-25); vii. UPEC+ginseng; viii. UPEC+CIPX (CIPX was started 48 hours after the UPEC injection) (23) and ix. UPEC+CIPX+ginseng.

### Preparation of Korean red ginseng

We initially prepared a suspension of white powder of ginseng root with 50% ethanol. The prepared suspension was boiled and condensed by vacuum, dried by speed vac. The resultant material was resolved with PBS (26). 

### Tissue and sample collection

At 14 days after the end of the experiment, all animals were anesthetized using ketamine (50 mg/ kg) and xylazine (12 mg/kg) and their prostate glands were carefully removed (10,23). 

### Histological analysis

All samples were fixed in 10% formalin and embedded in paraffin. Samples were sectioned by rotary microtome into 5 µm thick slices, stained with hematoxylin and eosin, and examined by light microscopy (Olympus DP25, Japan) using Image Analyzer software (ImageJ 1.43u). 

The severity of inflammatory cell infiltration, interstitial fibrosis and acinar changes, as indications of prostate inflammation were measured and graded on a scale from 0 to 5 (Table 1) (10,27). 

### Terminal deoxynucleotidyl transferase mediated dUTP-biotin nick end labeling assay

TUNEL was used to quantify apoptotic cells in the prostate epithelium. The procedure was performed according to recent studies (9). At the end of the staining, we evaluated the apoptotic index by counting the number of cells that showed TUNEL positivity in 100 cells each in 10 random slides from all groups by light microscopy at ×400 magnification (28,29). 

### Statistical analysis

Statistical analysis was performed using one way ANOVA followed by Tukey’s post hoc comparison test. The significance level was considered to be P<0.05.

**Table 1:** Severity scores of inflammatory cell infiltrations, acinar changes and interstitial fibrosis of prostate tissue


Score	Inflammatory cell infiltration	Acinar change	Interstitial fibrosis

0	No evidence	No evidence	No evidence
1	<10%	<10%	<10%
2	10-25%	10-25%	10-25%
3	25-50%	25-50%	25-50%
4	50-75%	50-75%	50-75%
5	75-100%	75-100%	75-100%


## Results

### Apoptosis

**Fig.1:**
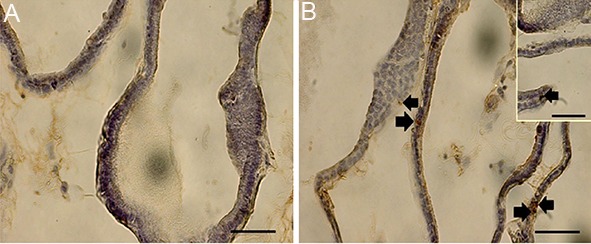
Analysis of apoptotic cells by TUNEL staining (×400). Arrows indicate apoptotic cells (scale bars; 50 μm). A. Control and B. Ciprofloxacin (CIPX). TUNEL; Terminal deoxynucleotidyl transferase mediated dUTP-biotin nick end labeling.

**Fig.2:**
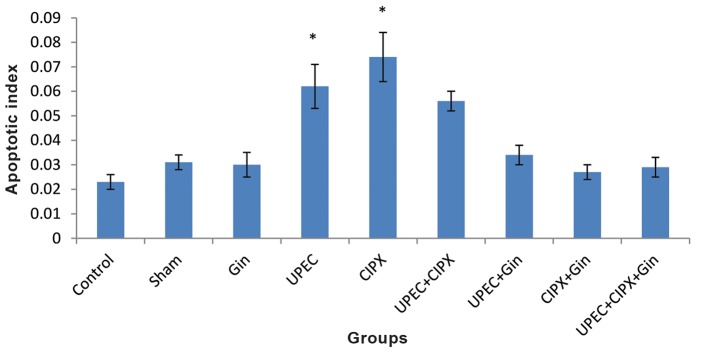
Histogram of apoptotic cell number. Note increased number of apoptotic epithelial cells in the CIPX, UPEC and UPEC+CIPX groups. Values represent the mean ± SEM number of TUNEL-positive cells per 1000 prostate epithelial cells. *; Significant difference at P<0.05 level compared with all groups except the UPEC+CIPX group, UPEC; Uropathogenic *Escherichia coli*, CIPX; Ciprofloxacin and Gin; Ginseng.

The number of TUNEL positive cells in the prostate epithelium greatly increased following antibiotic treatment (Fig.1). Figure 2 shows the mean apoptotic index in 1000 epithelial cells per group. The apoptotic index of both the CIPX and UPEC groups significantly increased in contrast with all other groups (P<0.05). The numbers of apoptotic cells in the UPEC+CIPX+ginseng group decreased compared to the UPEC+CIPX group. There was no significant difference between the UPEC+ginseng and control group (P<0.05). 

### Prostate histopathology

**Fig.3:**
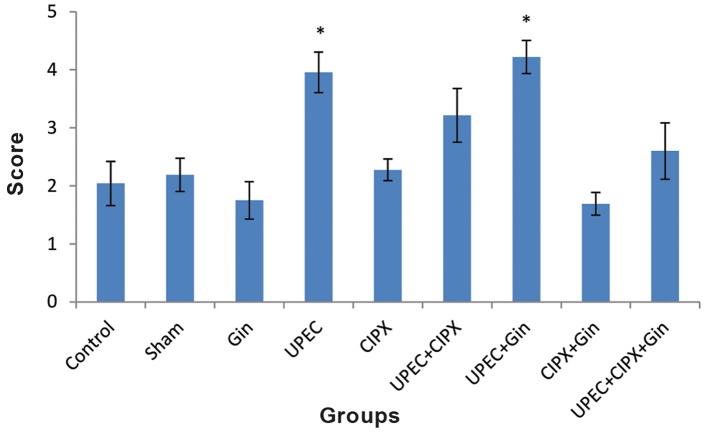
Severity scores for acinar changes. Data are presented as mean ± SEM. *; P<0.05 compared with all other groups (except UPEC+CIPX), UPEC; Uropathogenic *Escherichia coli*, CIPX; Ciprofloxacin and Gin; Ginseng.

**Fig.4:**
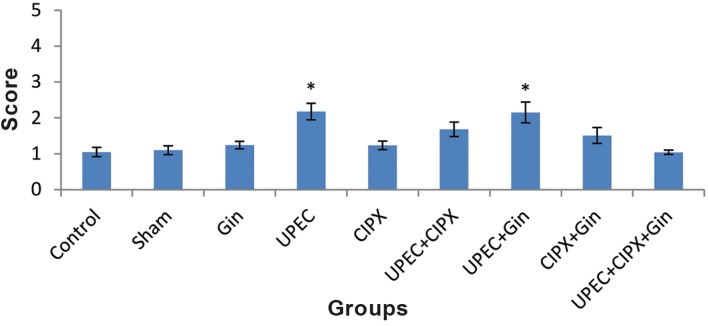
Severity scores for inflammatory cell infiltration. Data are presented as mean ± SEM. *; P<0.05 compared with all other groups except the UPEC+CIPX and CIPX+Gin groups, UPEC; Uropathogenic *Escherichia coli*, CIPX; Ciprofloxacin and Gin; Ginseng.

**Fig.5:**
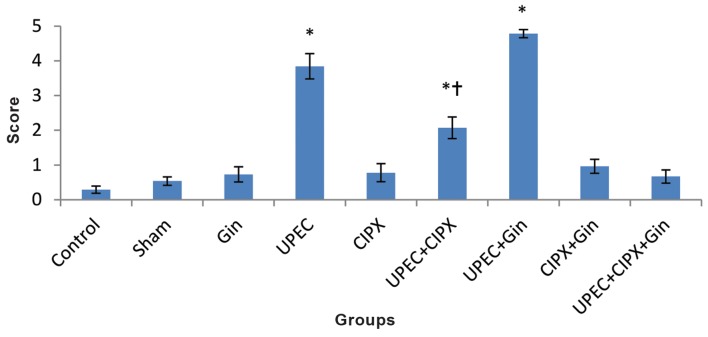
Severity scores for interstitial fibrosis. Data are presented as mean ± SEM. *; P<0.05 compared with all other groups, †; P<0.05 compared with the UPEC and UPEC+Gin groups, UPEC; Uropathogenic *Escherichia coli*, CIPX; Ciprofloxacin and Gin; Ginseng.

Histopathological investigation of the prostate was performed to evaluate acinar changes, inflammatory cell infiltration and interstitial fibrosis. Severity scores for these items in each group are given in Figures 3-5. Lymphocytes, monocytes and neutrophils comprised the majority of inflammatory cells.

**Fig.6:**
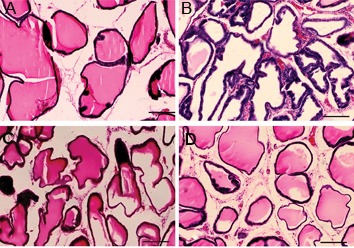
Prostate histological structure. A. Control group. The acinar structures are normal. There are a few marked inflammatory cell infiltrations and fibrosis in the interstitial space, B. UPEC. Acinar structures are folded and infiltration of inflammatory cells into the interstitial space is clearly seen, C. UPEC+CIPX. Note decreased numbers of inflammatory cells. Shrinkage of acinar structures decreased compared to the UPEC group and D. UPEC+CIPX+ginseng. Acinar structures returned to normal conditions. Mild inflammatory cell infiltration is seen. (H&E staining, magnification: ×100, scale bar: 200 μm). UPEC; Uropathogenic *Escherichia coli*, and CIPX; Ciprofloxacin.

Severity scores for acinar changes and inflammatory cell infiltration in the UPEC and UPEC+ginseng groups did not significantly differ (P<0.05), whereas scores of both groups increased significantly compared to the control group (Figes.3, 4). A comparison between the CIPX, CIPX+ginseng and control groups showed no significant differences in scores. This result was also in line with the interstitial fibrosis evaluation (Fig.5). There was no significant difference between the UPEC+CIPX+ginseng group and control group for all inflammatory items (Fig.6). The severity score of the UPEC+CIPX+ginseng group in contrast to the UPEC group showed a significant difference (P<0.05). Interstitial fibrosis evaluation showed significant increases in the UPEC, UPEC+ginseng and UPEC+CIPX groups compared with all other groups (P<0.05).

## Discussion

This study evaluated the protective effect of KRG on UPEC induced prostatitis in a rat model treated with CIPX. Our findings in the histopathological evaluations demonstrated no significant differences between the UPEC and UPEC+ginseng groups. In addition, the combined use of ginseng and CIPX was more effective than their single use. CIPX significantly increased the number of apoptotic cells. The anti-apoptotic effect of ginseng caused significant reductions in severity scores in the UPEC+CIPX+ginseng, UPEC+ginseng and CIPX+ginseng groups compared to the UPEC and CIPX groups. 

More than 50% of couples’ infertility problems are related to male factors (1,2). Among these, infections of the accessory sex glands such as prostatitis play an important role (6). For prostate infections, the use of antibiotics such as CIPX is the gold standard of treatment (10). However, recent studies report adverse effects of testicular dysfunction, DNA damage and chromatin abnormalities of sperm cells, and increased numbers of apoptotic germ cells in seminiferous tubules in males treated with CIPX (8,9,30). In addition, CIPX can impair the histological structure of the epididymis, testicles, seminal vesicles and prostate (11). These adverse effects can be related to increased numbers of ROS during CIPX treatment (14). Therefore, during this condition the use of a potent antioxidant can be helpful. KRG is one of the mostly used herbal medicines in East Asia (31). Ernst reported possible mechanisms of action as its anti-apoptotic effect, anti-inflammatory action, antioxidant, reduction of platelet adhesion and vasodilation (32). The anti-aging, anti-diabetic and anti-cancer effects of ginseng are also reported (33). 

Elkahwaji et al. (34) have shown interstitial edema, acute inflammatory cell infiltration and acinar shrinkage in prostate infected by *E. coli*. This finding agreed with our results which showed that the UPEC group significantly differed from the other groups in all inflammatory items. The observed increase in the UPEC group score could be attributed to excessive ROS production by leukocytes present in inflammatory conditions. According to our findings, interstitial fibrosis evaluation of CIPX treatment of UPEC induced prostatitis showed a significant difference compared to the UPEC group. This result confirmed findings by Kim et al. (27). On the other hand, there was no significant difference between the UPEC+CIPX and UPEC groups in inflammatory cell infiltration and acinar changes. Demir et al. (23) reported that CIPX used for treatment of *E. coli* infected testicles degenerates germinal epithelium. A possible explanation for these differences might be due to CIPX suppression of *E. coli* by blocking bacterial DNA synthesis (8). Therefore, CIPX reduces infection outcomes on histological structures. In addition, this antibiotic decreases serum testosterone levels (35,36) and indirectly affects male reproductive organs (23,37). 

Kim et al. (17) reported a protective effect of KRG on rat testicular dysfunction by suppression of ROS production. Choi et al. (18) suggested ginseng+CIPX to be an effective treatment in rats. Kim et al. (27) reported the good effect of ginseng combined with CIPX under inflammatory conditions. Our study supported recent studies by regarding the protective effect of ginseng. In the current study, there was no significant difference between the UPEC and UPEC+ginseng groups. Of note, the severity score of the UPEC+CIPX+ginseng group showed a significant decrease compared to the UPEC group in all inflammatory items. Ginseng could not suppress *E. coli* but it could eliminate excess ROS produced during antibiotic treatment. 

The UPEC and CIPX groups significantly increased apoptosis in prostate epithelial cells compared to the control group. Dwyer et al. (38) in a molecular assessment, showed that *E. coli* exhibited characteristic markers of apoptosis. Khaki et al. (9) reported an increased number of apoptotic germ cells per seminiferous tubules in the CIPX group compared to the control group. Nguyen et al. (39) and Kim et al. (40) proved the anti-apoptotic effect of KRG in neuroblastoma cells. To the best of our knowledge, there has been no specific study of the anti-apoptotic effect of ginseng on infected prostate epithelium. We have observed a significant difference between the UPEC and UPEC+ginseng groups, as well as between the CIPX and CIPX+ginseng groups. CIPX suppresses *E. coli* action by blocking bacterial DNA synthesis, however it increases the number of oxidants produced (8,14). According to our results, it can be concluded that ginseng is a good antioxidant to be used to eliminate excess oxidants. Therefore use of this antioxidant during oxidative stress conditions may be helpful. 

## Conclusion

These findings enhanced our understanding of anti-inflammatory and anti-apoptotic effects of KRG. Our experimental results have suggested that combined use of CIPX and KRG in UPEC infected rats can be helpful in treating male UTI and possibly improve fertility. These results are subject to certain limitations, such as measurement of serum testosterone levels and microbiological analyses. Further studies should be carried out in humans. 

## References

[1] Najafi G, Nejati V, Shalizar Jalali A, Zahmatkesh E (2014). Protective role of royal Jelly in oxymetholone-induced oxidative injury in mouse testis. IJT.

[2] Vaziri MH, Sadighi Gilani MA, Kavousi A, Firoozeh M, Khani Jazani R, Vosough Taqi Dizaj A (2011). The relationship between occupation and semen quality. Int J Fertil Steril.

[3] Choi GY, Cho JH, Jang JB, Lee KS (2004). Effects of panax ginseng on the sperm motility and spermatogenesis in the SD rat. Korean J Orient Med.

[4] Lang T, Dechant M, Sanchez V, Wistuba J, Boiani M, Pilatz A (2013). Structural and functional integrity of spermatozoa is compromised as a consequence of acute uropathogenic E.coli-associated epididymitis. Biol Reprod.

[5] Keck C, Gerber-Schäfer C, Clad A, Wilhelm C, Breckwoldt M (1998). Seminal tract infections: impact on male fertility and treatment options. Hum Reprod Update.

[6] Golshani M, Taheri S, Eslami G, Suleimani Rahbar AA, Fallah F, Goudarzi H (2006). Genital tract infection in asymptomatic infertile men and its effect on semen quality. Iranian J Public Health.

[7] Hryckowian AJ, Welch RA (2013). RpoS contributes to phagocyte oxidase-mediated stress resistance during urinary tract infection by Escherichia coli CFT073. MBio.

[8] Elias A, Nelson B (2012). Toxicological effect of ciprofloxacin on testicular function of male guinea pigs. Asian J Exp Biol Sci.

[9] Khaki A, Heidari M, Ghaffari Novin M, Khaki AA (2008). Adverse effects of ciprofloxacin on testis apoptosis and sperm parameters in rats. Iranian J Reprod Med.

[10] Lee YS, Han CH, Kang SH, LEE SJ, Kim SW, Shin OR (2005). Synergistic effect between catechin and ciprofloxacin on chronic bacterial prostatitis rat model. Int J Urol.

[11] Abu-Aita NA, Ahmed KA, Mouneir SM (2011). The protective effect of ginger and N-acetyl cysteine on ciprofloxacin-induced reproductive toxicity in male rats. J Am Sci.

[12] Tremellen K (2008). Oxidative stress and male infertility--a clinical perspective. Hum Reprod Update.

[13] Goswami M, Mangoli SH, Jawali N (2006). Involvement of reactive oxygen species in the action of ciprofloxacin against Escherichia coli. Antimicrob Agents Chemother.

[14] Goswami M, Mangoli SH, Jawali N (2007). Effects of glutathione and ascorbic acid on streptomycin sensitivity of Escherichia coli. Antimicrob Agents Chemother.

[15] Aksoy H, Yapanoglu T, Aksoy Y, Ozbey I, Turhan H, Gursan N (2007). Dehydroepiandrosterone treatment attenuates reperfusion injury after testicular torsion and detorsion in rats. J Pediatr Surg.

[16] Fanaei H, Azizi Y, Khayat S (2013). A review: role of oxidative stress in male infertility. JFUMS.

[17] Kim YH, Kim GH, Shin JH, Kim KS, Lim JS (2010). Effect of korean red ginseng on testicular tissue injury after torsion and detorsion. Korean J Urol.

[18] Choi YS, Cho YH, Han CH (2007). Synergistic effect between Ginsenoside or Urovaxom® with ciprofloxacin on chronic bacterial prostatitis rat model. Korean J Urol.

[19] Oremosu AA, Arowosaye VO, Akang EN, Bassey RB (2013). Effects of Cissus populnea and Panax ginseng on flutamide-induced testicular defect in pre-pubertal male rats. Br J Med Med Res.

[20] Sohn DW, Han CH, Jung YS, Kim SI, Kim SW, Cho YH (2009). Anti-inflammatory and antimicrobial effects of garlic and synergistic effect between garlic and ciprofloxacin in a chronic bacterial prostatitis rat model. Int J Antimicrob Agents.

[21] Kim SH, Ha US, Lee HR, Sohn DW, Lee SJ, Kim HW (2011). Do Escherichia coli extract and cranberry exert preventive effects on chronic bacterial prostatitis?. Pilot study using an animal model. J Infect Chemother.

[22] Eskandari M M, Jani S, Shokri S, Zeiyghami H, Yazdaninajad A (2014). Protective effects of chinese red ginseng on
the spermatogenic cells apoptosis and sperm quality of
epididymo-orchitis in the rat model.

[23] Demir A, Türker P, Onol FF, Sirvanci S, Findik A, Tarcan T (2007). Effect of experimentally induced Escherichia coli epididymo-orchitis and ciprofloxacin treatment on rat spermatogenesis. Int J Urol.

[24] Jantos C, Altmannsberger M, Weidner W, Schiefer HG (1990). Acute and chronic bacterial prostatitis due to E.coli.Description of an animal model. Urol Res.

[25] Kumar V, Prabha V, Kaur S, Kaur K, Singh SK (2011). Receptor dependent immobilization of spermatozoa by sperm immobilization factor isolated from Escherichia coli: proof of evidence. Int J Urol.

[26] Liou CJ, Li ML, Tseng J (2004). Intraperitoneal injection of ginseng extract enhances both immunoglobulin and cytokine production in mice. Am J Chin Med.

[27] Kim SH, Ha US, Sohn DW, Lee SJ, Kim HW, Han CH (2012). Preventive effect of ginsenoid on chronic bacterial prostatitis. J Infect Chemother.

[28] Colecchia M, Frigo B, Del Boca C, Guardamagna A, Zucchi A, Colloi D (1997). Detection of apoptosis by the TUNELtechnique in clinically localised prostatic cancer before and after combined endocrine therapy. J Clin Pathol.

[29] Pannek J, Berges RR, Sauvageot J, Lecksell KL, Epstein JI, Partin AW (1999). Cell turnover in human seminal vesicles and the prostate: an immunohistochemical study. Prostate Cancer Prostatic Dis.

[30] Zobeiri F, Sadrkhanlou RA, Salami S, Mardani K, Ahmadi A (2012). The effect of ciprofloxacin on sperm DNA damage, fertility potential and early embryonic development in NMRI mice. Vet Res Forum.

[31] Kang KS, Kim HY, Pyo JS, Yokozawa T (2006). Increase in the free radical scavenging activity of ginseng by heat-processing. Biol Pharm Bull.

[32] Ernst E (2010). Panax ginseng: an overview of the clinical evidence. J Ginseng Res.

[33] Vayghan HJ, Ghadimi SS, Nourazarian AR (2014). Preventive and therapeutic roles of ginseng-focus on colon cancer. Asian Pac J Cancer Prev.

[34] Elkahwaji JE, Zhong W, Hopkins WJ, Bushman W (2007). Chronic bacterial infection and inflammation incite reactive hyperplasia in a mouse model of chronic prostatitis. Prostate.

[35] Zobeiri F, Sadrkhanlou RA, Salami S, Mardani K (2013). Long-term effect of ciprofloxacin on testicular tissue: evidence forbiochemical and histochemical changes. Int J Fertil Steril.

[36] Khaki A, Sohrabi Haghdoust I, Ghaffari Novin M, Azarmi Y, Heidari M (2014). Effect of ciprofloxacin in rat spermatogenesis. ISMJ.

[37] Khaki A, Ghaffari Novin M, Khaki AA, Fathiazad F, Khabiri M, Hossinchi J (2009). Ultra structural study of gentamicin and ofloxacin effect on testis tissue in rats: Light and transmission electron microscopy. Afr J Pharm Pharmacol.

[38] Dwyer DJ, Camacho DM, Kohanski MA, Callura JM, Collins JJ (2012). Antibiotic-induced bacterial cell death exhibits physiological and biochemical hallmarks of apoptosis. Mol Cell.

[39] Nguyen CT, Luong TT, Kim GL, Pyo S, Rhee DK (2015). Korean Red Ginseng inhibits apoptosis in neuroblastoma cells via estrogen receptor β-mediated phosphatidylinositol-3 kinase/Akt signaling. J Ginseng Res.

[40] Kim EH, Lee MJ, Kim IH, Pyo SN, Choi KT, Rhee DK (2010). Anti-apoptotic effects of red ginseng on oxidative stress induced by hydrogen peroxide in SK-N-SH cells. J Ginseng Res.

